# Diagnostic Peptide Discovery: Prioritization of Pathogen Diagnostic Markers Using Multiple Features

**DOI:** 10.1371/journal.pone.0050748

**Published:** 2012-12-14

**Authors:** Santiago J. Carmona, Paula A. Sartor, María S. Leguizamón, Oscar E. Campetella, Fernán Agüero

**Affiliations:** 1 Instituto de Investigaciones Biotecnológicas, Instituto Tecnológico de Chascomús (IIB-INTECH), Universidad Nacional de San Martín, Consejo de Investigaciones Científicas y Técnicas (UNSAM-CONICET), Sede San Martín, San Martín, Buenos Aires, Argentina; 2 Instituto de Microbiología y Parasitología Médica, Facultad de Medicina, Universidad de Buenos Aires, Buenos Aires, Argentina; Federal University of São Paulo, Brazil

## Abstract

The availability of complete pathogen genomes has renewed interest in the development of diagnostics for infectious diseases. Synthetic peptide microarrays provide a rapid, high-throughput platform for immunological testing of potential B-cell epitopes. However, their current capacity prevent the experimental screening of complete “peptidomes”. Therefore, computational approaches for prediction and/or prioritization of diagnostically relevant peptides are required. In this work we describe a computational method to assess a defined set of molecular properties for each potential diagnostic target in a reference genome. Properties such as sub-cellular localization or expression level were evaluated for the whole protein. At a higher resolution (short peptides), we assessed a set of local properties, such as repetitive motifs, disorder (structured vs natively unstructured regions), trans-membrane spans, genetic polymorphisms (conserved vs. divergent regions), predicted B-cell epitopes, and sequence similarity against human proteins and other potential cross-reacting species (e.g. other pathogens endemic in overlapping geographical locations). A scoring function based on these different features was developed, and used to rank all peptides from a large eukaryotic pathogen proteome. We applied this method to the identification of candidate diagnostic peptides in the protozoan *Trypanosoma cruzi*, the causative agent of Chagas disease. We measured the performance of the method by analyzing the enrichment of validated antigens in the high-scoring top of the ranking. Based on this measure, our integrative method outperformed alternative prioritizations based on individual properties (such as B-cell epitope predictors alone). Using this method we ranked 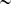
10 million 12-mer overlapping peptides derived from the complete *T. cruzi* proteome. Experimental screening of 190 high-scoring peptides allowed the identification of 37 novel epitopes with diagnostic potential, while none of the low scoring peptides showed significant reactivity. Many of the metrics employed are dependent on standard bioinformatic tools and data, so the method can be easily extended to other pathogen genomes.

## Introduction

Infectious diseases remain a major public health problem worldwide. Several intervention and control strategies have been devised throughout the years to manage these complex diseases. In this scenario, immunodiagnostics have been, and still are, essential tools for demonstrating infection, for follow up studies (clinical management, prognosis of a disease), and as tools to monitor success of control strategies, and to support infection surveillance campaigns [1]. Particularly in the case of intracellular pathogens, the most straight-forward strategies for immunodetection of pathogens usually rely on the detection of antibodies that bind to whole-parasite extracts or some fraction of a parasite, e.g. a flagellar fraction. These methods, however, suffer from specificity problems, as cross-reactive antibodies are common, confounding the diagnostic and often requiring additional (and perhaps more complex) diagnostic tests.

Development of new diagnostics is partly limited by the availability of well characterized antigens. Peptide scanning is a widely used technique for mapping linear epitopes in a protein antigen [2]–[5]. The recent availability of peptide microarray platforms allow rapid and inexpensive high-throughput serological screenings [6]. This, coupled with the increasing number of complete pathogen genomes, means that it is now theoretically possible to identify immunodominant linear epitopes by scanning all predicted protein sequences using a similar approach. For pathogens with small genomes – e.g. viruses and small bacteria – it is therefore straightforward to synthesize and test the presence of antibodies directed against thousands of individually addressable peptides, that in concert represent the whole proteome. However, this approach cannot be applied directly to bigger bacterial or eukaryotic genomes, given their larger proteomes. Therefore computational methods are required to filter down the list of candidate peptides to be tested, while at the same time enriching them in potentially reacting epitopes.

The challenge for this bioinformatic exercise is thus to identify, within a given proteome, those peptides that could be good targets for a B-cell response. The problem of B-cell epitope prediction, refers to the identification of regions in an antigen that are recognized by the corresponding binding site (“paratope”) of antibodies. Over time, a number of algorithms have been developed for the computational prediction of B-cell epitopes. [7]–[15] However, perhaps with the exception of immunodominant epitopes, the set of epitopes recognized by a polyclonal sera is not independent of the method of immunization (e.g. artificial immunization *vs.* natural infection), immunized species, use of adjuvants, etc. As a consequence, prediction of diagnostic epitopes in the context of a particular disease or infection is a more complex problem, where many additional constraints apply, such as mechanism of entry of the infectious agent, expression pattern of parasite proteins (when, where, abundance) amongst others. All these additional variables affect the outcome of the immune response, and may explain the variability in responses observed, for example, against the same protein in different species [3].

A number of successful antigen discovery efforts have been published recently, in which a computational strategy guided the selection of candidates for experimental validation. In *Trypanosoma cruzi* (a unicellular protozoan), Goto Y et al [16] identified and experimentally validated 8 antigens by searching for proteins bearing large tandem repeats; Cooley and coworkers [17] performed a high-throughput serological screening of *T. cruzi* proteins, prioritizing their candidates by known expression in relevant lifecycle stages, proteomic evidence and secretion or surface exposure likelihood. In this latter study, and starting from 400 proteins expressed in an heterologous system, the authors identified 39 promising antigens for further testing, and selected 16 for a multi-bead assay. In *Echinococcus* (a metazoan) List *et al.* described a bioinformatic filtering strategy, where they targeted alpha helical coiled-coils and intrinsically unstructured regions in secreted or surface-exposed parasite proteins [18]. Starting from 11 proteins from two *Echinococcus* species they identified 45 candidate peptides between 24 and 30 amino acids in length that were then screened using peptide microarrays. These papers provide a proof of principle for the discovery of diagnostically relevant large peptides using a computational selection.

However, we argue that many additional criteria can be integrated and exploited in a computational strategy to further guide the process of diagnostic peptide discovery. Firstly, we consider that there are significant advantages in using a peptide-level prioritization, as opposed to a protein selection process followed by peptide selection. Furthermore, we propose a feature weighting approach, in contrast to a strict filtering strategy that excludes targets/peptides that don't match the specified criteria.

For this exercise, we chose to use the genome of the protozoan parasite *Trypanosoma cruzi*, the causative agent of Chagas Disease, for a number of reasons. Firstly, the genome size of *T. cruzi* is large and complex for a protozoan parasite. Furthermore, this is an interesting biological model for the application of a diagnostic peptide discovery strategy, not only due to its high health impact and the need for novel diagnostics [19], but also because many antigens have already been described which can be used either to identify predictive features or to assess our method's efficacy.

Chagas disease is endemic in 18 countries in Central and South America, affecting up to 8 million individuals [20]. Vectorial transmission of the disease occurs in endemic countries through the bite of some hematophagous insects, or by consumption of food exposed to secretions from infected insects [21]. However, in non-endemic countries transmission mother-to child, blood transfusion, and organ transplantation also occurs. Diagnosis of the disease is challenging, because *T. cruzi* human infection evolves into a chronic stage where circulating parasites or their products are difficult to detect. In addition, serological diagnostic tests can be misleading due to cross-reactivity with other related protozoan pathogens that are geographically overlapped, such as *Leishmania spp.* (causative agent of Leishmaniasis) and *T. rangeli* (a south American trypanosome that does not cause disease). Currently, a “conclusive” diagnosis of *T. cruzi* infection is reached only after multiple serological tests [19], and there are urgent needs to develop new diagnostics that can be used in the early detection of congenital infections, to monitor blood banks and drug treatments in clinical studies.

In this work we present a comprehensive computational strategy for the discovery of diagnostically relevant peptides that can be applied to large genomes. We demonstrate the utility of our method by predicting candidate diagnostic epitopes starting from a complete eukaryotic genome.

## Results and Discussion

### Devising a computational strategy for diagnostic peptide prioritization

The main driving idea behind this computational exercise was to learn from known validated antigens and use this knowledge to find new candidate diagnostic markers. However, although the number of known antigens in our case study is reasonable (Chagas Disease, 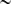
30 validated antigens), it is still a limited dataset to perform an unbiased learning exercise (e.g. using a machine-learning type of algorithm or a regression analysis). Therefore we decided to implement a prioritization strategy in which the selection of features and their weights was done manually (and may be subjective to some extent).

Ideally, as a result of this strategy we would like to end up with peptides containing continuous (linear) B-cell epitopes from an immunogenic protein that is also expressed by the pathogen during infection in a human host.

One other major constraint that our method had to take into consideration was derived from our choice of experimental screening platform. In our case, we decided to perform high-throughput B-cell epitope discovery and mapping experiments. Therefore, peptide microarrays were a natural choice mostly because of their good benefit-cost ratio, in comparison with, e.g. protein microarrays. Peptide microarrays display short peptides (usual length is 10–20 residues) deposited or directly synthesized on a solid support. As in phage display, these arrays reveal the binding of pathogen-directed antibodies to linear peptides that are either present in a similar linear conformation in the parent protein, or that mimic structural features of the epitope [22].

Based on these initial ideas and requirements, we started to define desirable and undesirable properties or features for inclusion in our computational biomarker discovery method. These are shown in Table 1. The table lists some properties that are assessed at the whole-protein level (e.g. protein expression, abundance) and others that are assessed at the level of short peptides (e.g. antigenicity, internal aminoacid repeats) or even at the level of individual residues (presence of non-synonymous polymorphisms). These will be described to some extent next.

**Table 1:** Features, Attributes and Tools.

Feature	Basis	Method	Weight (Score)
Cellular Surface Localization Index (CSLI)	Potentially secreted/surface protein	SignalP, DGPI	Positive, Large (5)
Protein Expression Index(PEI)	Timing and abundance of expression	Proteomic data, Codon Usage Bias, Gene copy number	Positive, Large (5)
Predicted B-cell epitopes	Antigenicity	Bepipred	Positive, Medium (3)
Internal Aminoacid Repeats	Immunogenicity	Trust	Positive, Medium (3)
Extracellular domain of integral membrane protein	Surface Localization	TMHMM	Positive, Low (1)
Trans-membrane domain	Low accessibility	TMHMM	Negative, Large (−5)
Natively unstructured region	Selection of linear epitopes	IUPred	Positive, Medium (3)
High local sequence similarity agains host proteins	Low immunogenicity	FASTA	Negative, Large (−5)
High local sequence similarity against related pathogens	Misleading diagnosis	FASTA	Negative, Large (−5)
Protein has an additional domain not present in other orthologs	Potential immunogenic domain	BLASTP/Perl	Positive, Large (5)
Potentially glycosylated regions	Avoid post-transcriptional modifications	NetOGlyc	Negative, Low (−1)
Regions with very low sequence complexity	Low specificity	SEG	Negative, Large (−5)
Region upstream of Signal peptide cleavage residue	Absent in mature protein	SignalP	Negative, Large (−5)
Region downstream of GPIanchor addition residue	Absent in mature protein	DGPI	Negative, Large (−5)
Intra-species genetic diversity(polymorphic residues)	Non conserved peptides	TcSNP Database	Negative, Large (−5)
Cysteine in peptides	Synthetic peptides are sensitive to oxidation and cyclization	Custom Perl Script	Negative, Large (−5)

The Table lists features evaluated by our computational pipeline, the basis for their selection and method for calculation. The numerical weight (score) listed for each features is applied to modulate the contribution of each attribute to the final peptides scores.

#### Assessment of the exposure of candidate antigens during an infection

The first properties we considered in our method were related to the expression, abundance, and subcellular localization of the proteins carrying the identified antigenic epitopes. Intuitively, a diagnostic epitope should be present in an abundant protein located either at the pathogen's surface, or secreted during the initial stages of infection; therefore maximizing early exposure of the protein to B-cells in the host. The utility of these criteria for selecting candidate antigens has been demonstrated recently by Liang *et al.* using full proteome microarrays containing *Brucella melitensis* proteins [23].

Abundance of pathogen proteins was assessed using a number of complementary strategies and datasets. From a diagnostics perspective, relevant proteins are those expressed by the life cycle stages of the pathogen that occur in the mammal host (in the case of *T. cruzi* these are the trypomastigote and amastigote stages). Proteomic data is available for all *T. cruzi* life cycle stages (successfully exploited for diagnostic marker discovery by Cooley *et al.* [17]). These data allows the identification of mass spectra (corresponding to peptides obtained after proteolytic digestion) and their relative abundance. However, the main drawback of these data is their low coverage. After grouping allelic copies and paralogues, we were able to obtain trypomastigote/amastigote expression (and abundance) information for only 649 proteins (7.5% of the predicted proteome, includes proteins with at least 1 mass spectra) or 349 (4% of the predicted proteome, when considering proteins with at least 2 mass spectra from at least 2 distinct peptides).

An alternative approach to estimate and compare expression levels for the entire proteome, takes advantage of evidence showing that gene copy number and codon usage are correlated with protein expression levels in trypanosomes [24]. Due to the limited gene regulation at the transcriptional level [25] it has been suggested that synonymous codon usage may have an important role in controlling protein abundance in trypanosomatids. Using a set of highly expressed tandem-repeated genes that displayed a strong synonymous codon usage bias, Horn has shown that there is a significant correlation between this biased codon usage and protein abundance in *T. cruzi* [24]. Using the same dataset, we calculated a Codon Adaptation Index (CAI, see Methods) for all protein coding genes. This index is a measure of how similar the codon usage of a given gene is in comparison with the biased codon usage derived from the set of highly expressed genes. Therefore, it provides indirect evidence for protein abundance in any life stage. For the final scoring of protein abundance we derived a composite “protein expression index” (PEI) score that combines the proteomic data (counts of mass-spectra normalized over protein length), gene copies (paralogues) numbers, and CAI (see Methods).

The most probable subcellular localization of proteins was assessed using standard bioinformatic tools (see Table 1 and Methods). Using these tools, we identified proteins that contain classic ER-secretory route signals, membrane attachment signals (e.g. glycosylphosphatidyl inositol anchors), and trans-membrane domains. To prioritize diagnostic targets, we derived a composite “Cellular Surface Localization Index” (CSLI) based on these predictions (see Methods). Proteins with positive predictions for membrane/secretory route localization are usually synthesized in the cell as pre-proteins, that are later cleaved to remove N-terminal (ER or mitochondrial signal peptides) or C-terminal (trailing end after GPI anchor addition) peptides. These peptides are not present in mature protein products. Therefore, at the peptide level, we also used data from these predictions to penalize these regions in our peptide prioritization strategy (see “Integration” section below).

Other regions that, although present in a mature product, are probably not available for B-cell receptors are highly hydrophobic sequences (e.g. those located in buried trans-membrane domains) or those covered by post-translational modifications, such as those that are O-glycosylated. These regions were similarly identified and scored by our method (see Table 1 and Methods).

#### Predicting the potential antigenicity and immunogenicity of proteins and peptides

From the standpoint of a serological diagnostic method, our interest is focused on identifying markers that are able to elicit a strong antibody response. A number of predictive algorithms have been developed over time to identify antigenic regions of proteins from their primary structure based on aminoacid propensity scales [7], [26]–[30]. The performance of these original methods however, was marginally better than random [31]. Computational prediction of B-cell epitopes is still an active research field and a number of state of the art predictors show improved performance [8], [9], [11]–[15], however, prediction accuracies are still not satisfactory. Here we are interested in identifying B-cell epitopes recognized by naturally infected humans, which could represent a special subset of all known epitopes. However, current B-cell epitope predictors are trained on epitopes derived from heterogeneous experimental conditions (e.g. the AntiJen dataset [32]) including many cases in which laboratory animals were immunized with relatively large doses of highly purified antigens. It has been described that humoral responses against the same antigen can differ between species (human, rat, mouse, dog, rabbit, etc.) [3], [33] but also between members of the same species (significant variability in individual B-cell epitopes reactivity has been reported in pulmonary tuberculosis and toxoplasmosis [34], [35]). This variability may be explained for example, by the genetic background of the immunized host, and its immunological memory (previous exposure to different antigens). Despite all these drawbacks, state of the art B-cell epitope predictors are indeed capturing some signals shared by these heterogeneously determined epitopes. For example, immunodominant epitopes, “seen” across different species and experimental conditions, could represent a dataset with such shared features.

In our bioinformatic method, we include results of predictions from one state-of-the-art B-cell epitope predictor (Bepipred) [8]. However, based on our assessment of its performance we gave a moderate weight to its overall contribution to our own prioritization method (see Table 1).

#### Aminoacid tandem repeats as surrogate markers of immunogenicity

Another feature that contributes to a proteinâ's immunogenicity is the presence of tandem repeats, defined as two ore more copies of an aminoacid sequence. Particularly in the case of tandemly-repeated, short, aminoacid sequences, it has been demonstrated that the overall immunogenicity of proteins harboring these repeats is increased, as well as the antigenicity of epitopes contained within these repetitive units [36]–[39]. In the case of *T. cruzi* many of the currently validated protein antigens are repetitive. SAPA (Shed Acute Phase Antigen) is the repetitive C-terminal domain of a number of members of the trans-sialidase superfamily [3]; the Surface Antigen 2 (CA-2) is another well validated antigen that is composed almost entirely of imperfect repeats [40]. The presence of tandem repeats as an indicator of the potential immunogenicity of proteins was validated to some extent in *T. cruzi* in previous works. Out of 9 repetitive proteins assayed, Goto and coworkers showed that 8 of these were recognized specifically by sera from *T. cruzi* infected patients [16].

#### Prioritizing linear peptides, and exploiting sequence similarity to avoid non-immunogenic and cross-reactive epitopes

To select the best candidate peptides for our choice of experimental screening platform, we decided to prioritize epitopes located in intrinsically unstructured regions. Natively unfolded (disordered) regions are ubiquitous across species, but are particularly abundant in eukaryotes, participating in a wide range of functions [41]. We expect that peptides in these regions would display similar conformations both in the native protein and in the form of short peptides immobilized on a solid support. Therefore we gave a moderate positive weight to peptides that were located in the context of intrinsically unstructured regions.

Furthermore, because intrinsically unstructured regions lack well defined 3D structure, primary sequence similarity might predict, more than in any other case, the potential for molecular mimicry of a peptide, not only against host proteins, but also to avoid selecting potentially cross-reacting epitopes. Due to the immunological tolerance, peptides with high sequence similarity to host proteins are expected to be non-antigenic (to avoid self recognition) [42]. Similarly, sequence similarity between two pathogens could result in the identification of cross-reactive markers. Therefore we gave large negative weights to peptides that showed high similarity against human and *Leishmania* peptides (see Table 1 and Methods), and to very low complexity sequences (e.g. homopolymers). The approach is essentially similar to that followed to build the mimicDB Molecular Mimicry database [43].

One other similarity-based feature that we incorporated in our method, was the identification of natural chimeric proteins in which one part of the protein represents a conserved region/domain, while another part corresponds to a region uniquely found in the parasite (a phylogenetically restricted domain). A number of such cases were described for *T. cruzi*, corresponding to two major antigens, SAPA/trans-sialidase, and cruzipain [44]. In these cases a conserved catalytic domain (neuraminidase-like, and cathepsin-like, respectively) is attached to a unique region, that may be repetitive (as in the SAPA antigen) or non-repetitive (as in the C-terminal domain of cruzipain), but that was found to be highly immunogenic, directing the immune response away from the catalytic domains. In our strategy we searched for these cases using a similarity-based approach (see Methods).

### Integration of selected criteria for diagnostic target and epitope prioritization

Based on the assessment of the attributes described above, we obtained a fine grained map of peptide- and protein-level features for the *T. cruzi* proteome. These data were stored in a custom MySQL database, including sequence location of features and the corresponding prediction scores, allowing for fine granularity in the interrogation of the data.

For our diagnostic epitope discovery strategy, we followed a peptide-level prioritization/scoring approach. The output of our method is a ranked list of peptides, therefore facilitating the task of designing and organizing the peptides in an array. In this strategy, each peptide was rewarded or penalized with points, based on the presence of desirable or undesirable properties carried by the peptide itself, or by its parent protein. To calculate the final score for a peptide we first normalized individual peptide feature scores (the raw scores provided by each prediction tool) so that all scores fell a fixed 0–1 range (see Methods). A sigmoid transform was applied in many cases to reduce the influence of extreme values (seeFigure S1). Finally, features were manually assigned to six classes according to their *a priori* expected influence on their diagnostics potential: positive low effect, positive medium effect, positive large effect, negative low effect, negative medium effect and negative large effect, with associated numerical weights of 1, 3, 5, −1, −3 and −5, respectively (listed in Table 1). Once the normalized feature scores and weights were defined, the score for each peptide (PS) was simply calculated as:
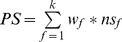
where *k* is the number of evaluated features, and *w_f_* and *ns_f_* are the weight and normalized score of feature *f*. By applying this strategy, we effectively ranked each of the 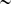
10 million 12-residue overlapping peptides in which our pathogen proteome can be conceptually fragmented.

**Figure 1:**
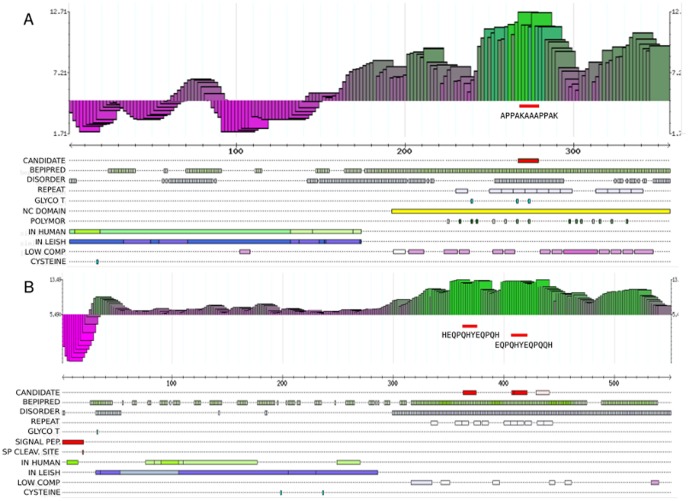
Visualization of peptide-score profiles generated by the method. A) the 60S ribosomal protein L19 (locus identifier TcCLB.509149.40), and B) a putative lectin (locus TcCLB.506239.30). These plots display peptide scores and features along protein sequences. Mapped features in these examples are those listed in Table 1: antigenicity (Bepipred), protein disorder, internal repeats, signal peptide, signal peptide cleavage site, non-synonymous polymorphisms, high conservation vs human, high conservation vs *Leishmania spp*, low sequence complexity, glycosylated threonines, cysteines, and presence of domain absent in orthologous proteins (NC DOMAIN). Vertical boxes represent overlapped 12-residue peptides, and their height and level of green are proportional to the peptide score. They vary around their base protein scores (i.e. 4.7 and 5.5), which accounts for subcellular localization and expression.

We generated a visualization of these data in the form of a peptide score plot with a number of feature-tracks aligned to the plot (see Figure 1). These were used to facilitate the task of manually locating and inspecting candidate or previously described epitopes within a complete protein. As observed in the Figure, peptides are displayed in the plots as colored boxes (width = 12 residues) with both the color and height of the boxes conveying information about the peptide's score. The feature tracks below the plot provide a simple way to rapidly locate interesting attributes as well as a visual aid when decomposing the score for any peptide. The Figure shows only a couple of examples, but profiles for all proteins have been generated and are available as supplementary material.

To our knowledge this approach is innovative, as previously reported diagnostic antigen discovery strategies were all protein-centric, narrowing protein candidates based on properties averaged over the protein sequence length, and on the presence of favorable features (protein disorder, antigenic propensity, low sequence similarity against host, etc) without consideration for how these features overlap. Therefore, our peptide-centric approach allows the identification of peptides with excellent characteristics but that don't overlap with otherwise unfavorable features. In previous strategies, proteins containing these undersirable features would have been penalized, and filtered at early stages of the selection process. In our case, in contrast, these proteins will not be removed from the analysis, and all peptides will also be assessed. A final score will be calculated for each peptide, that is the result of all the feature overlaps, and their corresponding weights.

### Testing the method: assessing enrichment of previously described antigens

**Figure 2:**
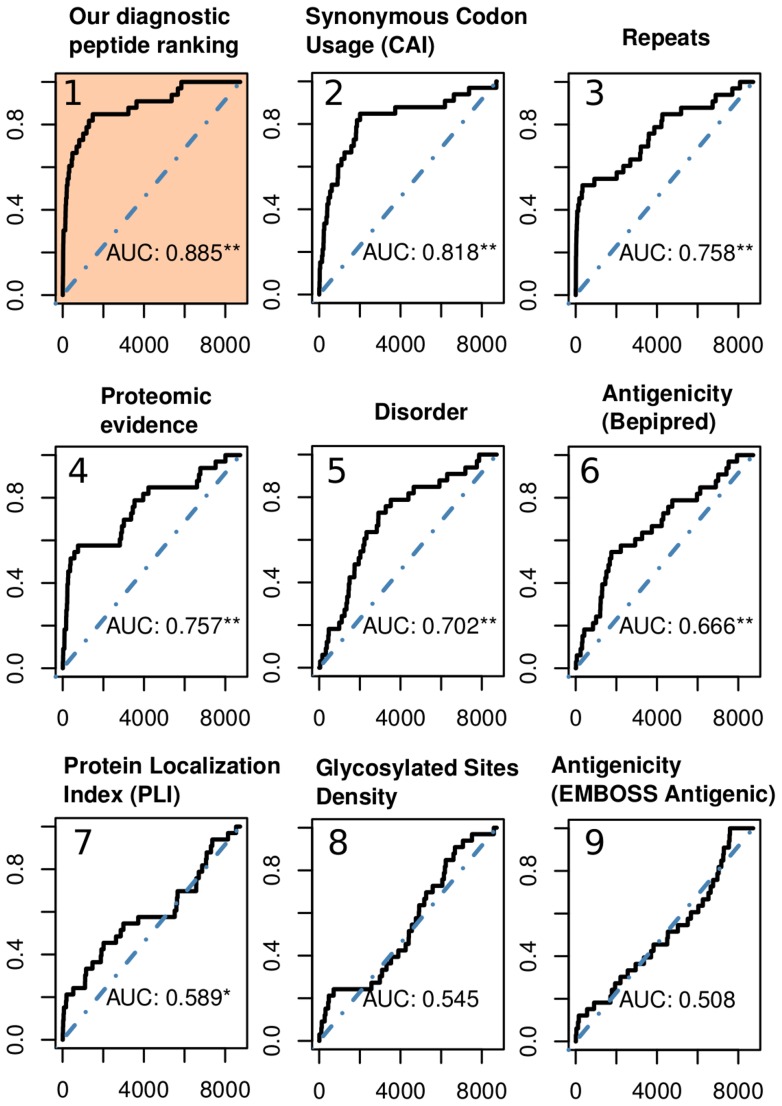
Assessing enrichment of known antigens. The figure shows a number of enrichment plots obtained under different prioritization scenarios. In all plots: the x axis contains the prioritized proteome (top ranking proteins at the origin); the y axis displays the fraction of known validated antigens recovered in the top x proteins; the blue dashed line displays an hypothetical enrichment plot with an AUC = 0.5 (expected by chance), while the black solid line represents the actual enrichment obtained in each prioritization. From the top-left: comparison of different prioritization strategies (ordered by decreasing AUC values): 1) our composite method, 2–9) a number of prioritizations using a single criteria in each case: 2) Codon Usage bias (CAI), 3) Internal repeats, 4) Proteomic evidence of expression, 5) natively unstructured regions, 6) antigenicity (Bepipred), 7) surface localization (GPI), 8) O-Glycosylation, 9) antigenicity (EMBOSS antigenic). 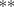 p-value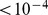, 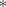 p-value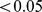 (p-values based on a random permutation test, n = 10,000).

To validate the strategy of epitope prioritization we analyzed the performance of our method by measuring its ability to rank known validated antigens. For this exercise we used a dataset of 33 non-homologous *T. cruzi* antigens compiled from the literature and the IEDB database as our set of validated antigens (Table S2). To produce a ranked list, we merged redundant information (data from paralogues and allelic copies of the same locus) into a set of 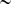
8,700 clusters. Using this dataset we produced a number of different prioritizations, for comparison purposes. These are analyzed in detail in Figure 2. In one case we calculated the overall score for each protein, as described above, as the summation of the individual weighted scores of all features (our composite method). The only difference in this case, was the omission of the analysis of potentially cross-reacting peptides (high local similarity against related pathogens such as *Leishmania*), mostly because the antigens in our test set were not tested for cross-reactivity. In all other cases, we used a single criteria or index to obtain a ranked list of proteins. In all cases the highest scoring member of each cluster was chosen to represent the group for the final ranking. Enrichment (recall) of known validated antigens was assessed simply by calculating the cumulative number of these antigens for each ranking position (black solid curve in each plot, see Figure 2). The resulting area under the curve (AUC) therefore represents a measure of enrichment.

As an example, ranking the clustered *T. cruzi* proteome using proteomic evidence of expression in amastigotes and trypomastigotes as the single prioritizing criteria, produces a highly significant enrichment (AUC = 0.757, Bonferroni corrected p-value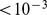, as estimated from 10^4^ random permutations, fourth plot in Figure 2). This finding is plausible with the idea that proteomic studies usually sample the most abundant proteins in an extract, and that these proteins are therefore more likely to be targets of the human humoral response. Other features that produced significant enrichment of known antigens are the use of a codon adaptation index to measure a codon usage bias similar to that found in highly expressed genes (AUC = 0.818, P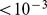) (a surrogate indicator of a potential for high levels of expression), the number of internal repeats in a protein (related to the immunogenicity of the protein, AUC 0.758, P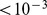), the presence of natively unstructured regions in proteins (AUC 0.702, P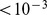), the antigenicity as predicted by Bepipred (AUC 0.666, P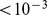), and the exposure of proteins at the cell surface (AUC = 0.589, P
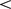
0.05). However, other criteria performed only marginally better or not better than a random ranking: *i.e.* they produced an AUC value close to 0.5. This is the case, as expected, for the epitope predictor Antigenic. This algorithm belongs to the family of predictors based on aminoacid propensity scales, which were recently shown to perform poorly [31].

As can be observed in the Figure, our composite method produced the highest enrichment (AUC = 0.885, corrected p-value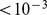) outperforming all other prioritizations. This is not surprising because our method integrates additional, orthogonal information on the prioritized antigens. Interestingly, the analysis of synonymous codon usage patterns, as measured by the CAI, yielded the highest performing individual feature. The CAI measures the similarity of a gene's synonymous codon usage against that found in a set of highly expressed genes, therefore serving as a surrogate marker for high levels of expression. In our case the CAI was calculated against a set of *T. cruzi* highly expressed genes (as measured by mass spectra counts). This means that the CAI index and the proteomic evidence are correlated. However, interestingly the CAI method outperformed the latter, presumably by identifying highly expressed proteins that were not detected in the mass spectrometry study.

The fact that our method can successfully recall 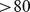
% of the known validated antigens from the top 20% entries in the ranking, essentially means that the currently known antigens share many features which could be collectively exploited to prioritize antigens. However, although this enrichment exercise provides support *a posteriori* for our prioritization method, this test dataset was not unbiased and big enough to be used in a reverse engineering strategy (e.g. a completely unbiased feature selection coupled with a feature weight optimization process).

### Experimental validation: selecting and testing candidate peptides in a peptide-chip format

Our ultimate goal was to identify novel diagnostic antigens, therefore at this stage we decided to further validate our method by experimentally testing peptides from the top of the ranking. To select peptides for inclusion in a peptide-chip, every protein in the predicted proteome was first assigned the score of its highest-scoring 12-mer peptide. Next, protein profiles (see Figure 1) were generated for the 300 top scoring proteins (large protein groups containing many paralogs were clustered, and one profile was only generated for the gene with highest score). These profiles were manually examined and in all cases we selected between 1 and 5 peptides for experimental validation (these are so far serologically uncharacterized peptides). As mentioned before, in this strategy we penalized peptides with many non-synonymous SNPs, as they are not conserved within the species and would favor the identification of peptides that may not be recognized by humans infected by different strains of the pathogen. Notwithstanding this, in a second prioritization, we only considered polymorphic peptides (number of non-synonymous SNPs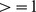), and now rewarded the presence of non-synonymous sites, reasoning that this could help us discover polymorphic peptides with the ability to serologically discriminate between distinct evolutionary lineages. In this alternative prioritization, these top scoring “polymorphic” peptides all had significantly lower scores than those in the main peptide prioritization. A second group of peptides was selected for inclusion in the array from this set, to provide a useful contrast of e.g. higher scoring vs. lower scoring peptides, and hopefully to identify lineage discriminating epitopes.

As a result of this selection, we obtained a set of 190 12-mer peptides (high-scoring) that were synthesized and spotted in glass slides (see Methods). These peptides, derived from 85 different protein products, were present in the microarray in three internal replicas, which also contained 36 additional synthetic peptides derived from curated, validated, B-cell linear epitopes, and other 40 peptides (low scoring) that contain a moderate to high degree of allelic polymorphism (these were derived from 16 pairs of polymorphic proteins). An exploratory experimental validation was conducted using these peptide-chips. Five slides were incubated with sera of Chagas Disease patients from different geographic areas in Argentina, 5 other arrays were incubated with sera of healthy donors and 2 were incubated with sera of Leishmaniosis patients (1 visceral leishmaniosis, 1 cutaneous leishmaniosis, both serologically negative for Chagas' disease.

**Figure 3:**
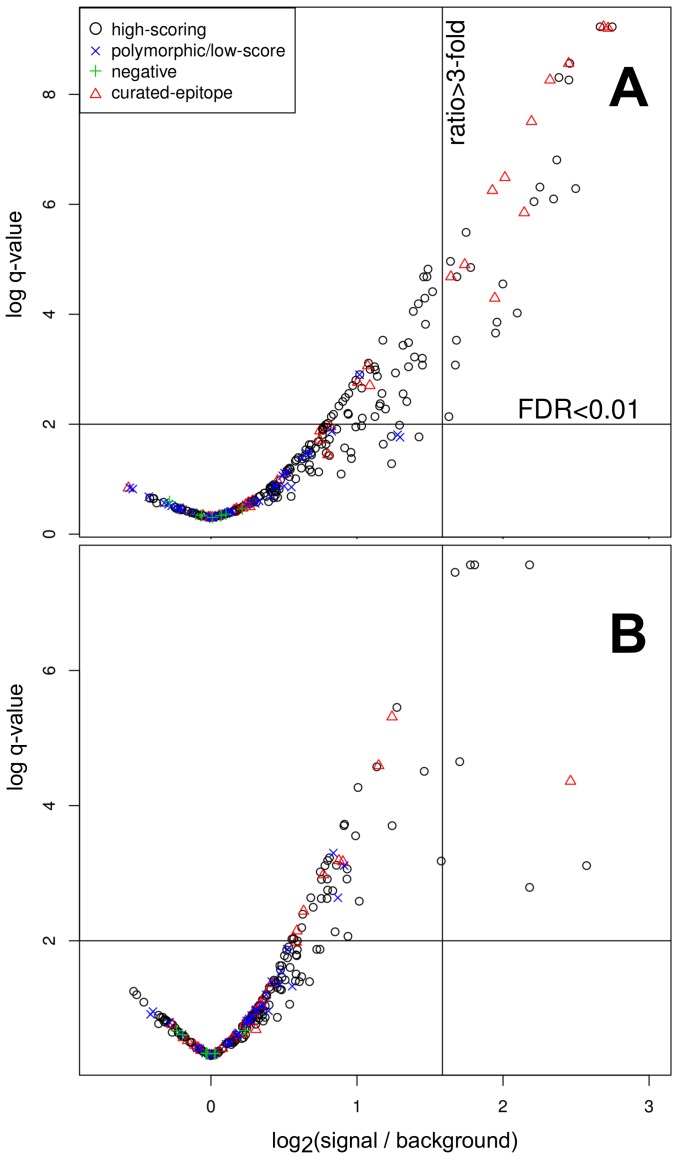
Experimental Distribution of peptide intensities ratios (log2 fold change) vs. statistical significance of the signal (negative log scale of q-value) after multiple testing adjustment. FDR = False Discovery Rate. The q-value is the FDR analog of the p-value. Panel A corresponds to measurements obtained from a peptide chip assayed with sera from a pool of Chagas positive samples, while panel B corresponds to a chip assayed with sera from healthy donors. Points in the higher-right corner of the quadrant are marked as reactive peptides.

Binding was quantified for each spot in the array, and statistically significant signals were determined as described in Methods. An example scanned image of an array is available in Figure S2. Plots of antibody-binding data for two representative arrays are shown in Figure 3).

The serological reactivity profiles obtained were in all cases consistent between replicates (within the 3 internal sub-arrays) but variable across individual positive samples, showing that epitope specificities differ between Chagas Disease patients. Notwithstanding the observed variability, there were a number of spots that consistently gave high intensity, and high signal/noise ratios against all sera tested. A detailed list of peptides, and their reactivities against different sera is available as Table S3. Amongst these, there were a number of peptides derived from currently validated antigens such as the TcD/Ag13 surface antigen [45], the JL8/CRA/R-27 antigen [46], the Ag2 (CA-2,B13) antigen [40], and the TcE antigen (ribosomal protein L19) [47]. Novel epitopes discovered in this exercise were derived from proteins that were not characterized as antigens previously. These are good candidate epitopes for further serological characterization. At the top of this list is a short peptide from an hypothetical protein (locus ID TcCLB.504159.10) that has 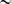
68% of its length covered by almost perfect repeats of a short, 14-aminoacid repeat (consensus sequence: “GGFGSATHTSAPAA”). In the array, the 12-mer peptide with the sequence “APAVGGFGSAAH” gave consistently high signal/background ratios, being recognized by 80% of the chagasic sera tested.

Other interesting peptides were those that showed a high-specificity profile against the panel of sera. In this case good candidates were those that gave significant reactivity with any of the positive sera for Chagas Disease, but not with any of the negative or Leishamaniosis sera. These conditions were satisfied by 32 of the 190 high-scoring selected peptides (16.8%, derived from 23 distinct novel proteins), 13 of the 36 curated epitopes (36.1%) and by none of the polymorphic (low scoring) peptides (0/40) (see Table 2). A complete listing of the peptides that were reactive against at least one sera, is available in Table 3 and as Table S1. These data suggest that the selection of high-scoring peptides: i) provides a signal that is infection-specific (as the number of reactive peptides in positive samples is larger than the number of peptides in samples from healthy subjects, Mann–Whitney–Wilcoxon test, 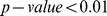); and ii) is enriched in B-cell epitopes (as the proportion of reactive peptides in this set is larger than the corresponding fraction in the polymorphic (low-scoring) peptide set (Fisher's Exact Test, 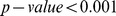).

**Table 2:** Summary of peptide reactivities.

Peptide Class	Assayed	AND Chagas (+)	AND Healthy (−)	AND Leishmaniasis (−)
Curated	36	16 (44.4%)	13 (36.1%)	13 (36.1%)
New	190§	52 (27.4%)	37† (19.5%)	32* (16.8%)

The table summarizes the results from the screening of pools of positive (Chagas), negative (healthy donors) and related (Leishmaniasis) sera. From left to right the columns show the results of cumulative additional criteria (boolean AND): 1) Assayed, 2) Assayed AND Positive for Chagas Disease sera, etc.

§ derived from 85 distinct proteins.

† derived from 27 distinct proteins.

* derived from 23 distinct proteins.

**Table 3:** Complete list of reactive peptides.

ID	Gene Name	Description	Pos.	Sequence	Score	Tc+ (N = 5)	Healthy+ (N = 5)	Leish+ (N = 2)
n42	TcCLB.508175.329	60S ribosomal protein L19, putative	335	PAKAAAAPAKAA	10.57	80%	0%	0%
n67	TcCLB.509149.40	60S ribosomal protein L19, putative	275	APPAKAAAPPAK	12.84	80%	0%	0%
n126	TcCLB.504159.10	hypothetical protein, conserved	443	APAVGGFGSAAH	6.91	80%	0%	0%
n86	TcCLB.508831.150	hypothetical protein, conserved	47	SPFKSVFGAPSS	7.30	60%	0%	50%
n90	TcCLB.506239.30	lectin, putative	409	EQPQHYEQPQQH	13.54	60%	0%	0%
n96	TcCLB.511671.50	hypothetical protein, conserved	47	ESPFKSVFGAPS	7.10	60%	0%	0%
n25	TcCLB.510305.70	hypothetical protein, conserved	457	FPVVGMPRPGGF	8.91	60%	20%	50%
n1	TcCLB.511633.79	microtubule-associated protein, putative	239	DVGPRHVDPDHF	11.48	40%	0%	0%
n26	TcCLB.510305.70	hypothetical protein, conserved	463	PRPGGFPVVGMP	8.62	40%	0%	0%
n38	TcCLB.508175.329	60S ribosomal protein L19, putative	233	AAAAPAKAAAAP	10.68	40%	0%	0%
n40	TcCLB.508175.329	60S ribosomal protein L19, putative	273	AKAATAPAKAAA	8.94	40%	0%	0%
n85	TcCLB.508831.150	hypothetical protein, conserved	41	EKPPAESPFKSV	8.15	40%	0%	0%
n87	TcCLB.508831.150	hypothetical protein, conserved	53	FGAPSSTAAKPP	7.73	40%	0%	0%
n88	TcCLB.506239.30	lectin, putative	363	HEQPQHYEQPQH	12.99	40%	0%	0%
n41	TcCLB.508175.329	60S ribosomal protein L19, putative	323	KAATAPAKAAAA	9.91	40%	20%	0%
n154	TcCLB.506441.20	hypothetical protein, conserved	677	ERSGREGRERGY	9.56	40%	20%	50%
n190	TcCLB.508719.70	hypothetical protein, conserved	390	RCRGVYAPKTGT	6.76	40%	20%	50%
n24	TcCLB.510305.70	hypothetical protein, conserved	451	MPRPGGFPVVGM	8.72	20%	0%	50%
n28	TcCLB.506177.20	lectin, putative	347	QYEQPQQHYEQP	12.75	20%	0%	0%
n31	TcCLB.506177.20	lectin, putative	393	QPQQHEQPQQYE	12.94	20%	0%	0%
n44	TcCLB.508385.10	hypothetical protein, conserved	1313	GQYSPQHPQWNA	8.00	20%	0%	0%
n51	TcCLB.506791.30	hypothetical protein, conserved	1775	DPGPPVPAFTFA	7.34	20%	0%	0%
n56	TcCLB.510217.10	hypothetical protein	95	SPEPSAAWRNFA	9.23	20%	0%	0%
n63	TcCLB.506559.559	antigenic protein, putative	2209	RQPFVLPEPQET	10.35	20%	0%	0%
n74	TcCLB.506959.90	hypothetical protein, conserved	123	TAPAAPEPPRTA	9.46	20%	0%	0%
n77	TcCLB.508677.60	hypothetical protein	99	RGGPCPPNPAPP	11.68	20%	0%	0%
n89	TcCLB.506239.30	lectin, putative	407	HYEQPQHYEQPQ	13.49	20%	0%	0%
n97	TcCLB.511671.50	hypothetical protein, conserved	53	VFGAPSSTAAKP	7.34	20%	0%	0%
n112	TcCLB.508595.20	UDP-Gal-dependent glycosyltransferase	41	GAPGPNNPRHPR	11.76	20%	0%	50%
n115	TcCLB.506147.190	hypothetical protein, conserved	253	PSKPSPKAAPKK	9.82	20%	0%	0%
n122	TcCLB.510565.11	tyrosine aminotransferase, putative	27	KPSPSPKPIIKL	7.98	20%	0%	0%
n124	TcCLB.510733.50	hypothetical protein, conserved	99	KPSPKAAPKKAM	10.02	20%	0%	50%
n129	TcCLB.510877.40	hypothetical protein, conserved	173	RGGRGGGRGNNS	12.13	20%	0%	0%
n135	TcCLB.511861.120	hypothetical protein	97	PRPCVPDGGPTD	9.85	20%	0%	0%
n136	TcCLB.511861.120	hypothetical protein	101	VPDGGPTDVWTG	10.26	20%	0%	0%
n147	TcCLB.504625.70	kinetoplast DNA-associated protein, putative	443	VAREEAARRMHE	9.56	20%	0%	0%
n152	TcCLB.506441.20	hypothetical protein, conserved	665	RGYPEEKEDSRR	9.87	20%	0%	50%
n161	TcCLB.503975.100	hypothetical protein, conserved	343	AGPYGGMGGNGA	7.02	20%	0%	0%
n165	TcCLB.507603.260	cathepsin L-like, putative	353	APGPSPSYFVQM	11.65	20%	0%	0%
n184	TcCLB.463155.20	retrotransposon hot spot (RHS) protein	511	PRVLIGTPGIGN	7.56	20%	0%	0%
n186	TcCLB.511815.170	hypothetical protein, conserved	50	KEEVPEEVNAPE	10.09	20%	0%	0%
n176	TcCLB.511233.20	60S ribosomal protein L34, putative	111	HAKSQKEKKRRD	10.75	20%	20%	0%
n183	TcCLB.511345.10	retrotransposon hot spot (RHS) protein	539	FPLVDGFFFVDT	4.52	20%	20%	50%

Curated (validated) epitopes§								
ID	Gene Name	Description	AA Pos.	Sequence	Score	T. cruzi+ (N = 5)	Healthy+ (N = 5)	Leishmania+ N = 2
c1	TcCLB.505975.20	TcD/Ag13	903	KPAEPKSAEPKP	13.10	80%	0%	0%
c2	TcCLB.505975.20	TcD/Ag13	905	AEPKSAEPKPAE	13.10	80%	0%	0%
c10	TcCLB.506303.80	R27-2/FRA/JL8	1145	ATKVAEAEKQKA	8.23	60%	0%	0%
c12	TcCLB.506303.80	R27-2/FRA/JL8	1153	KQKAAEATKVAE	8.24	60%	0%	0%
c7	TcCLB.508831.140	B13/Ag2/CA-2	217	DKPSPFGQAAAG	8.66	40%	0%	0%
c8	TcCLB.508831.140	B13/Ag2/CA-2	589	GKPSPFGQAAAG	8.24	40%	0%	0%
c19	TcCLB.506659.10	Tc40	409	AKAAAPPAAARS	3.97	40%	0%	0%
c11	TcCLB.506303.80	R27-2/FRA/JL8	1149	AEAEKQKAAEAT	8.27	20%	0%	0%
c17	TcCLB.508355.250	60S acidic ribosomal protein P0	311	EDDDDDFGMGA	8.07	20%	0%	0%
c24	TcCLB.503419.30	thiol-transferase Tc52^*^	71	SRYIDRISSPAN	1.73	20%	0%	0%
c28	TcCLB.509391.30	Flagellar calcium-binding protein 3^*^	33	RIRQAIPREKTA	7.63	20%	0%	0%
c35	TcCLB.507511.91	TSSA	39	ENKPATGEAPSQ	6.88	20%	0%	0%
c36	TcCLB.507511.91	TSSA	41	KPATGEAPSQPG	7.29	20%	0%	0%
c22	TcCLB.503419.30	thiol-transferase Tc52	41	PQWYKELNPRET	3.89	20%	20%	50%
c27	TcCLB.509391.30	flagellar calcium-binding protein 3^*^	27	RKEAWERIRQAI	6.97	20%	20%	50%

Peptides displaying at least one *T. cruzi* positive assay with at most 1 healthy individual (control) positive are listed, showing the corresponding Locus Identifier, protein description, amino acid start position, sequence, prioritization score and the percentage of assayed samples in which the peptide was positive for *T. cruzi* infected, healthy control and *Leishmania* infected subjects (e.g. peptide n42 reacted in 4 of 5 -80%- of the *T. cruzi* samples). Letters n and c in Peptide ID indicate “novel” (highly ranked) and “curated” peptides, respectively.

§ Bibliographic references for validated antigens can be found in Table-S2, except for antigens marked with ^*^.

From a more general perspective, it is worth pointing out that although the bioinformatics strategy was guided by a rational selection of features derived from knowledge of previously described antigens, only 8 out of the 85 proteins represented in the array had significant sequence similarity to any of 33 previously characterized *T. cruzi* antigens (BLASTP vs the complete *T. cruzi* proteome; E-value
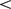
0.01). Furthermore, none of the succesful peptides in the experimental screening were derived from these 8 proteins. This shows that even though the proteins containing high-scoring peptides might have a feature space that is similar to that of previously known antigens, they could not have been selected using simple sequence similarity searches.

### Conclusion

We have described an integrated approach for diagnostic B-cell epitope discovery. This strategy allowed us to prioritize all 12-mer peptides from the complete proteome of a complex pathogen such as *Trypanosoma cruzi* for inclusion in a high-throughput screening platform. A first serological screening using short-peptide microarrays allowed the identification of new epitopes with diagnostic potential. Further serology characterization of these peptides is required to obtain a thourough diagnostic profile (in terms of their sensitivity and specificity) of these candidates. We conclude that peptide microarrays combined with a bioinformatic peptide selection strategy constitute a powerful and cost-effective platform for serodiagnostic biomarker screening of infectious diseases caused by pathogens with large and complex proteomes. The method described can be easily extended to other pathogen genomes.

## Methods

### Datasets and bioinformatic analysis

*T. cruzi* genome data was obtained from the TriTrypDB/GeneDB databases [48], [49]. Natively unstructured regions were identified with IUPred [50] (“short” type disorder with default parameters). For B-cell epitope/antigenicity prediction, we used BepiPred 1.0 [8] with default parameters, and EMBOSS Antigenic with window length = 6 [7], [51]. Trust 1.0 [52] was used to detect protein internal repeats, with the substitution matrix BLOSUM80 and low complexity filtering inactivated. Subcellular localization signals were assessed with SignalP 3.0 [53] (signal peptide), DGPI [54] (Glycosylphosphatidylinositol anchor), TMHMM 2.0 [55] (trans-membrane domains). To predict putative O-glycosylated residues we used NetOGlyc 3.1 [56]. Low complexity sequences were detected with SEG [57] (windows length = 6, low cut-off = 1 bit). Grouping of paralogous genes and putative allelic copies in the *T. cruzi* genome was based on the ortholog detection pipeline implemented by the OrthoMCL database [58], [59]. Some large gene families such as the trans-sialidase superfamily, mucins, mucin-associated proteins (MASP) and dispersed gene family protein 1 (DGF-1), initially assigned to multiple OrthoMCL gene clusters, were merged according to the their current annotation and sequence similarity. Information on non synonymous SNPs between allelic copies of *T. cruzi* genes were obtained from the TcSNP Database of *T. cruzi* genetic variation [60]. To calculate genome-wide Codon Adaptation Indexes, we used EMBOSS CAI [51], [61]. The codon usage table was generated with EMBOSS CUSP, from a set of *T. cruzi* highly expressed tandem repeated genes compiled by Horn [24]. Conserved aminoacid stretches between the target proteome and other potential cross-reacting species or the host species were detected by calculating small local sequence alignments using FASTA 3.4 (gap opening and extension penalties, 10 and 30 respectively; ktup = 2; substitution matrix = BLOSUM80) [62]. To detect non-conserved domains between orthologues, pathogen proteins were aligned against human proteins using BLASTP. Significant reciprocal best matches (E-value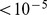) were filtered to identify cases were: i) at least 50% of the human protein (including its C-terminal) and at least 50% of the pathogen protein were aligned; ii) the C-terminal unaligned region of the pathogen's protein is at least 30 residues longer than the human counterpart; and iii) the C-terminal unaligned region doesn't belong to a PFAM domain.

The dataset of experimentally tested *T. cruzi* antigens used for the enrichment analysis was compiled from the literature [16], [17], [40], [45], [63] and from the Immune Epitope Database [64] (search for peptidic B-cell epitopes from source organism *T. cruzi* and host species *Homo sapiens*). Identified peptidic epitopes were then mapped to the corresponding protein using EMBOSS *fuzzpro*, allowing for 1 missmatch every 10 residues (mismatches can be due to sequence polymorphisms—).

Output from all programs was parsed with custom Perl scripts, and transformed into protein features, that were loaded into a MySQL database in GFF format (storing feature name, gene id, position in protein and score).

### Peptide scoring, ranking and visualization

#### Scoring of peptide features

Protein sequences were scanned to identify all overlapping 12-residue peptides in which the protein can be conceptually fragmented. At each step, the MySQL database was queried (see above) to retrieve information for the features mapped to each 12-mer peptide. For each feature, a raw feature score (RFS) was calculated as 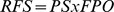, where PS (prediction score) is the score assigned by the software package used to make the prediction, and FPO (feature-peptide overlap) was calculated as the fraction of the peptide length covered by the feature (e.g.: if a 12-mer peptide is part of a trans-membrane domain that begins in its 6th residue, its feature-peptide overlap is 50%). Each raw (peptide) feature scores was stored in the MySQL database, mapped to the corresponding location coordinates (begin, end) of the peptide in the parent protein. Although the total number of possible 12-mer overlapping peptides is 9,540,317, we noticed that the scores of contiguous peptides (1 residue offset, 11 residues overlap) were highly correlated. Therefore, to accelerate the process, we decided to calculate scores only for peptides starting at odd positions, effectively producing a 2 residue offset, and a 10 residue overlap between contiguous peptides, and reducing the total number of peptides by half.

#### Normalization of peptide scores

To normalize the peptide feature scores, a transformation was applied to obtain values for all features that fit into a 0–1 range. In cases of features that showed distribution of scores with extreme values, a sigmoid transform was applied. For example, this was the case for the number of repetitive motifs in proteins, or the number of paralogous copies of a gene. The function used (SNF, Sigmoid Normalization Function) was:
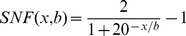
where *x* is the unscaled score and *b* is a scaling parameter. For each feature, we set *b* to the 99 percentile of its unscaled score. Therefore, when *x* equals *b*, SNF(x,b) approaches 0.9. As a consequence, when the unscaled feature score is in the top 1% of the distribution, the normalized score would fall in the range 0.9–1 (i.e. it is an outlier robust normalization). An example of this normalization is available in Figure S1 A. In all cases percentiles were calculated from homologous gene clusters (not using all genes), as large gene families distort the ranking. For cases of feature scores with flat distributions, no complex transformations were necessary. In these cases the normalized score was simply calculated as the ratio of the peptide score and the maximum value for the feature. Examples of these cases ere the assessment of natively unstructured, and low complexity regions, the Codon Adaptation Index, and SignalP Scores (peptide and cleavage). Normalization of sequence similarity scores was performed with a different criterion, to obtain a transformed measure of similarity that penalizes highly similar peptides (e.g. to avoid cross-reacting epitopes) and then rapidly decays in a non linear fashion. This re-scaled similarity (RS) score was defined as: 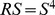 where *S* is the alignment sequence identity. As an example, a score of 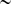
0.5 was obtained with this function for a sequence identity of 84% (S = 0.84). This value then grows rapidly until 100% sequence identity (see Figure S1 B).

In a few cases where more than one metric/feature are indicators of a common biological property (e.g. protein expression or subcellular localization) we used composite scores. For protein localization we defined a composite scoring function (Cellular Surface Localization Index, CSLI) based on feature scores from SignalP (signal peptide) and DGPI (GPI anchor):
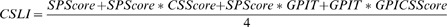
where SP = Signal Peptide; CS = signal peptide Cleavage Site; GPIT = GPI hydrophobic Tail presence (binary); GPICS = GPI Cleavage Site. All these scores where scaled by dividing over their maximum values. For the assessment of protein expression, we defined a composite scoring function (Protein Expression Index, PEI) based on proteomic data (mapped mass spectra), the codon adaptation index (CAI) and the number of copies of the gene in the genome. In this case the calculation of this PEI score uses the sigmoid normalization function (SNF) defined above:
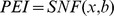


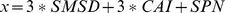


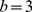
where SMSD = Scaled MSD (Mass Spectra Density); CAI = Codon Adaptation Index; SPN = Scaled Paralog Number; MSD = Mass spectra count/protein length. Again, these scores where scaled by dividing over their maximum values.

### Human specimens

Human serum samples were obtained from an endemic area in Argentina. *T. cruzi* infection was evaluated by ELISA based on epimastigote lysates, ELISA based on recombinant antigens (Wienner, Rosario, Argentina), Indirect Hemoaglutination Assay (Laboratory Polychaco, Buenos Aires, Argentina), and Indirect Immunofluorescence assay (IFI). Samples from patients reacting in two serological tests were scored as infected.

Leishmaniasis infection was evaluated using ELISA based on parasite lysates and Indirect Immunofluorescence assay (IFI). Visceral leishmaniosis was also evaluated using an rK39-based immunochromatographic test. All samples were negative for *T. cruzi* infection.

Samples from healthy donors were negative for both *T. cruzi* and *Leishmania spp*. Serum samples were stored at −20°C until use.

### Ethics statement

This study was conducted in accordance with the Declaration of Helsinki. Written informed consent was obtained from all donors. This study was approved by the Ethical Comittee of the Instituto de Investigaciones Biotecnológicas, Universidad Nacional de San Martín. Research did not involve interaction with the serum donors nor their identification.

### Serological screening

Peptide microarrays (JPT Peptide Technologies, Berlin, Germany) were employed in the serological screening. Twelve array slides were assayed with the following samples: 5 slides with pools (3 subjects per pool) of sera from patients infected with *T. cruzi*; 5 slides with pools (3 subjects per pool) of sera from healthy donors; 1 slide with a pool of 5 patients suffering from visceral leishmaniasis and 1 slide with a pool of 5 patients with cutaneous leishmaniasis. Serum samples were diluted at 1/100 with TBS-blocking buffer (Sigma, St. Louis, Mo) and incubated with sample dilutions at 30°C for 2 hs in a humidity chamber. After washing with TBS buffer, secondary anti-total human immunoglobulin G conjugated to Cy5 (Jackson ImmunoResearch), was diluted (1 µg/ml) in TBS buffer (Sigma, St. Louis, Mo) and added, and slides were incubated for 30 min at 30 in a humidity chamber. Finally, the slides were washed several times, including a final washing step with deionized water. Slides were dried by centrifugation before reading in the scanner.

### Quantification and Analysis of peptide reactivities

Slides were scanned using fluorescence readers GE Typhoon and ArrayWorx BioChip Reader at maximal resolution (pixel size 25 µm and 10 µm respectively). Laser excitation wavelength was 633 nm (red) and an emission filter of 670 nm (band pass 30) was used, according to the secondary antibody fluorophore. Digital array images were analyzed and peptide spots intensities were quantified with the software GE ImageQuant with no background subtraction ( 1% of the spots signals were discarded from the analysis as they presented bad quality signals due to fluorescence smearing, spikes or other forms of non-homogeneous spot intensities). The average intensity for each peptide was calculated from sub-array triplicates. All sub-arrays carry 4 spots of human IgG attached to the glass slides as positive controls. These spots were positive in all slides assayed. Also, all slides contain a number of spots where human and mouse proteins (beta-casein, human and mouse IgM, etc.) were attached to the slides by the manufacturer of the array to test them as candidate negative controls. However, these showed the same variability across slides as other test peptides, probably due to cross-reaction issues. Therefore we selected a set of 6 peptide spots with consistently low signal across all samples to estimate the background distribution. To identify statistically significant and biologically meaningful reactive peptides, we calculated the intra-chip fold change of peptide spots (3 replicates average) relative to the backgroud set average and tested mean differences (multiple Student's T-tests). Peptide spots with intensities bigger than 3 fold and with a False Discovery Rate 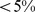 (Benjamini and Hochberg's FDR method) were considered positive. This filter allows the identification of peptides with statistically significant signal in each slide, however, it does not take into account the source of the sera used to assay each slide (infected patients, healthy individuals). Further filtering is possible if this additional information is taken into account (see Results).

## Supporting Information

Figure S1
**Examples of feature score scaling/transformation.** **A:** Sigmoid normalization of the score for aminoacid tandem repeats (motif copies). This feature score was normalized using the sigmoid function (SNF) described in Methods, with b = 12 (99th percentile) so that when a peptide bears a motif repeated 12 times, its feature score becomes 0.9. This transformation function scales the feature score into a 0–1 range, and is outlier robust. **B:** Non-linear, fourth-power scaling of the sequence identity of peptides against the proteome of *Leishmania* (potential cross-reacting pathogen). The same function was used to score similarity against the human (host) proteome. The function (see Methods) strongly penalyzes identities close to 1 (e.g. a 12-mer peptide sharing 11 residues with the target protein would produce a normalized score of 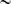
0.71 ).(PDF)

Figure S2 **Annotated image of a sector of an array.** The figure shows one sector (out of three identical sectors) of a slide assayed with Chagas positive sera (A), and one sector from a slide assayed with sera from a healthy donor (B). These slides are different than those used in Figure 3. Annotations include the descriptions of proteins from which the peptides in the array were derived. Positive controls (human IgG spots) correspond to whole proteins spotted on the glass. Colored boxes group spots with similar annotation/origin. Not all marked/annotated spots in this sub-array sector passed subsequent quality tests using data from the three internal replicas. Figure available in file: Figure S2.pdf
(PDF)

Materials S1 **Selected peptide profiles.** Two sets of protein peptide-score profiles, are included as supplementary materials, for the purposes of visualization of prioritized peptides. The plots are explained in Figure 1. A Peptide score profiles for the high-scoring proteins included in the arrays. Additional information on the selected peptides can be found in Table S3. B Peptide score profiles for the top 1000 protein candidates (ranked by their highest scoring peptide). For clusters of orthologous genes, only the best candidate of the cluster is considered.
(ZIP)

Table S1 **Complete list of reactive peptides.** Peptides displaying at least one *T. cruzi* positive assay with at most 1 healthy individual (control) positive are listed, showing the corresponding Locus Identifier, protein description, amino acid start position, sequence, prioritization score and the percentage of assayed samples in which the peptide was positive for *T. cruzi* infected, healthy control and *Leishmania* infected subjects (e.g. peptide n42 reacted in 4 of 5 -80%- of the *T. cruzi* samples). Letters n and c in Peptide ID indicate “novel” (highly ranked) and “curated” peptides, respectively. Data available in File: Table S1.xls
(XLS)

Table S2 **List of known (validated) antigens used to measure enrichment.** Data available in File: Table S2.xls
(XLS)

Table S3 **Complete list of peptides included in the array, showing the reactivity (1) or lack of reactivity (0) against different serum samples.** The criteria for considering a peptide reactive is similar to that shown in Figure 3. Data available in File: Table S3.xls
(XLS)
