# Supplementary material for: Diagnostic Peptide Discovery: Prioritization of Pathogen Diagnostic Markers Using Multiple Features
Source: PLoS One. 2012 Dec 14;7(12):e50748. doi: 10.1371/journal.pone.0050748 (PMC3522711; doi:10.1371/journal.pone.0050748)
Supplement: Figure S2 — Annotated image of a sector of an array. The figure shows one sector (out of three identical sectors) of a slide assayed with Chagas positive sera (A), and one sector from a slide assayed with sera from a healthy donor (B). These slides are different than those used in Figure 3. Annotations include the descriptions of proteins from which the peptides in the array were derived. Positive controls (human IgG spots) correspond to whole proteins spotted on the glass. Colored boxes group spots with similar annotation/origin. Not all marked/annotated spots in this sub-array sector passed subsequent quality tests using data from the three internal replicas. Figure available in file: Figure S2.pdf (PDF) [file pone.0050748.s002.pdf]

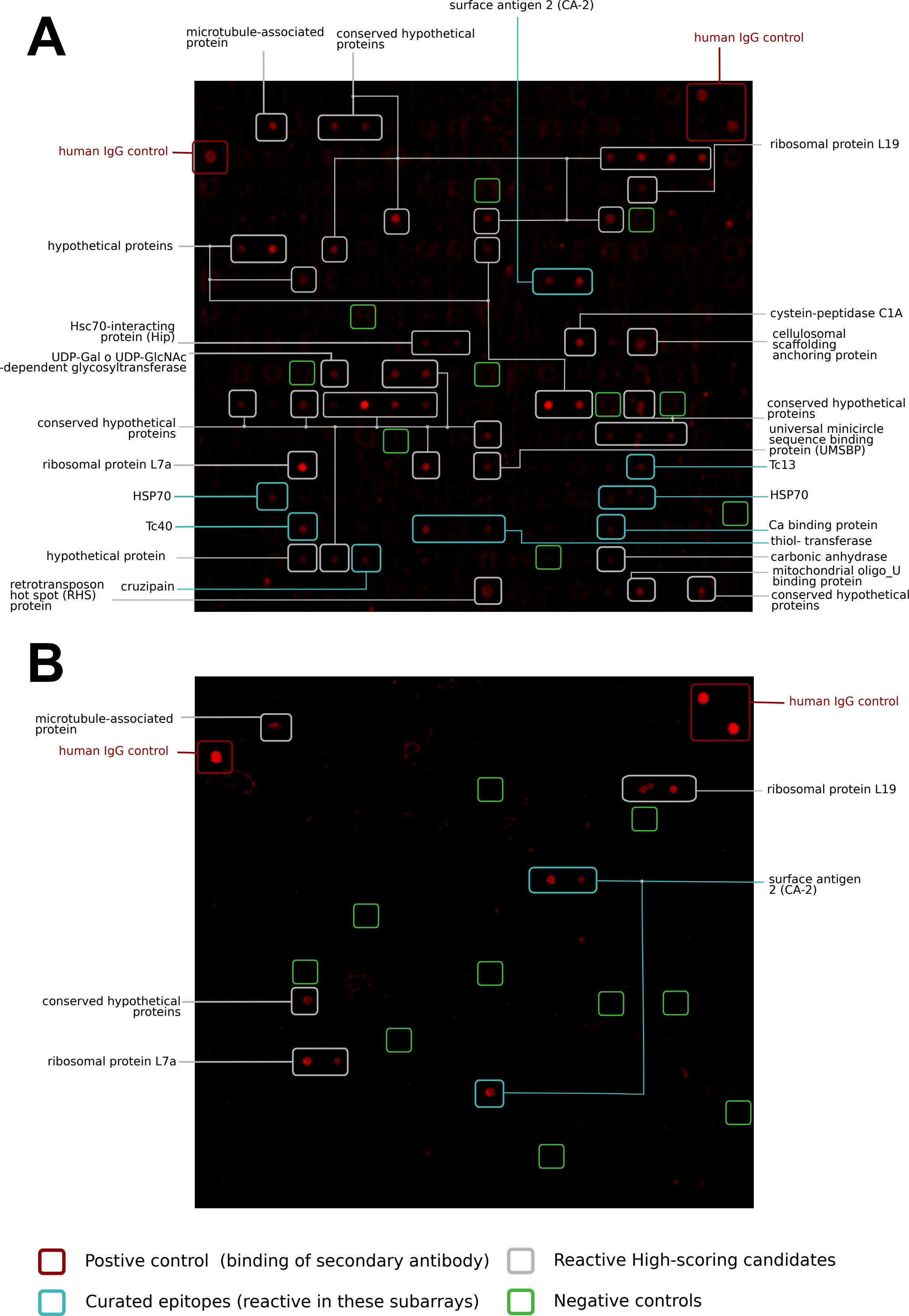
A
surface antigen 2 (CA-2)
microtubule-associated protein
conserved hypothetical proteins
human IgG control
human IgG control
ribosomal protein L19
hypothetical proteins
cystein-peptidase C1A
Hsc70-interacting protein (Hip)
cellulosomal scaffolding anchoring protein
UDP-Gal o UDP-GlcNAc -dependent glycosyltransferase
conserved hypothetical proteins
conserved hypothetical proteins
universal minicircle sequence binding protein (UMSBP)
ribosomal protein L7a
Tc13
HSP70
HSP70
Tc40
Ca binding protein
thiol- transferase
hypothetical protein
carbonic anhydrase
mitochondrial oligo_U binding protein
retrotransposon hot spot (RHS) protein
cruzipain
conserved hypothetical proteins
B
microtubule-associated protein
human IgG control
human IgG control
ribosomal protein L19
surface antigen 2 (CA-2)
conserved hypothetical proteins
ribosomal protein L7a
Postive control (binding of secondary antibody)
Reactive High-scoring candidates
Curated epitopes (reactive in these subarrays)
Negative controls
